# Mortality in Qatari individuals with mental illness: a retrospective cohort study

**DOI:** 10.1186/s12991-024-00499-w

**Published:** 2024-04-18

**Authors:** Sami Ouanes, Lien Abou Hashem, Ibrahim Makki, Faisal Khan, Omer Mahgoub, Ahmed Wafer, Omer Dulaimy, Raed Amro, Suhaila Ghuloum

**Affiliations:** Department of Psychiatry, Hamad Medical Corporation, POBOX 3050, Doha, Qatar

**Keywords:** Mortality, Mental illness, Death, Morbidity, Retrospective cohort

## Abstract

**Introduction:**

There is substantial evidence that people with mental illness have higher mortality rates than the general population. However, most of the studies were from Western countries, and it is not clear whether this finding also applies to Arab countries like Qatar.

**Objectives:**

We aimed to explore whether mortality in patients with mental illness in Qatar, is different from those without.

**Methods:**

We conducted a retrospective cohort study, including all Qatari nationals deceased in 2017 and 2018, using the list of registered deaths from Hamad Medical Corporation (HMC) Mortuary. We divided the cohort of deceased people into two groups: with and without mental illness. For each of the groups, we collected the age at death, the reported cause of death as well as sociodemographic and clinical data.

**Results:**

There were 602 registered deaths in 2017 and 589 deaths in 2018. The prevalence of mental illness was 20.4%. Compared to subjects without mental illness, subjects with mental illness surprisingly had higher age at death (median ± IQR = 76.5 ± 22.1 years vs. 62.7 ± 32.9 years; *p* < .001). This difference persisted even after we controlled for covariates. Individuals with mental illness were more likely to die of an infection (OR = 1.98[1.44;2.71]), or of chronic respiratory disease (OR = 3.53 [1.66;7.52]) but less likely to die because of accidental (OR = 0.21[0.09;0.49]) or congenital causes (OR = 0.18[0.04;0.77]).

**Conclusion:**

Contrary to most previous studies, we did not find that mortality was higher in Qatari individuals with mental illness. Sociocultural factors, free and easy-to-access healthcare, and an enhanced role of mental health professionals in detecting medical comorbidities may explain this finding.

## Introduction

There is substantial evidence that people with mental illness have higher mortality rates than the general population [1, 2]. A meta-analysis by Walker et al. published in 2015 found that 135 out of 148 studies examining mortality in people with mental disorders found significantly higher rates than controls. In the same meta-analysis, the pooled relative risk of mortality among those with mental disorders was 2.22. The estimated median number of years of potential life lost was about 10 [3].

This gap is probably increasing, as there is some evidence that patients with mental illness are not seeing the same improvement in life expectancy as the general population [4].

However, most studies about mortality in people with mental illness were carried out in Western countries, very few in Arab countries, and none, to our knowledge, in Gulf Council countries (GCC). In Tunisia, a study that examined mortality among inpatients at the main psychiatric hospital in the country between 2000 and 2010 found increased mortality (almost two-fold) among young adult inpatients when compared to the general population [5].

The increased mortality in people with mental illness has been attributed to an increase in both natural and unnatural deaths [2, 3, 6]. Suicide has been found to account for most of the elevated risk of unnatural death [2, 3, 7].

However, most deaths in patients with mental illness were natural. Cause-specific mortality rates have been found to be significantly increased for cardiovascular disease [2, 6–8], respiratory diseases [2, 6, 7], cancer [2, 6, 7], endocrine and metabolic conditions [2, 7], alcohol misuse [2], as well as infectious diseases [2, 9].

Among the leading mortality causes in patients with mental illness, cardiovascular events play an important role. In fact, cardiovascular mortality has been found to be increased among patients with most psychiatric conditions: schizophrenia [4, 7], schizoaffective disorder [7], bipolar disorder [7, 8], unipolar depression [7, 10], and anxiety disorders [11].

Moreover, cardiovascular mortality among people with mental illness is following an upward trend during the last years [7, 12], which makes cardiovascular disease one of the most important comorbidities to prevent, screen for, recognize and treat in patients with mental illness.

Major cardiovascular risk factors, namely smoking, metabolic syndrome, diabetes, hypertension, and dyslipdemia have been found to have a high prevalence in people with mental illness [7, 8, 11, 12]. Yet, people with mental illness are less likely to receive tobacco counseling [13], and less likely to be screened for cardiovascular disease, since they tend to develop cardiovascular events at an age younger than the age at which screening strategies are typically started in the general population [4]. Furthermore, the widespread use of second-generation antipsychotics may be contributing to further increasing the cardiovascular risk [1]. Even though a meta-analysis of the mortality outcomes in randomized, placebo-controlled trials of second-generation antipsychotics did not find evidence that second generation antipsychotics were associated with an overall increase in mortality in the wider clinical population, the same meta-analysis points out an increase in mortality in certain subgroups [14]. Moreover, most included trials were short-term, over a few weeks, which may have not allowed to actually capture the potential increase in cardiovascular mortality associated with atypical antipsychotics [1].

The increased cardiovascular mortality is possibly even more important in the GCC in general, and in Qatar in particular, where the prevalence of metabolic syndrome, diabetes, hypertension, and dyslipidemia is particularly high in the general population [15] as well as among people with mental illness [16].

In Qatar, all deaths transit through Hamad Medical Corporation (HMC) Mortuary. Even though expatriates may die and be buried abroad, Qatari nationals are always repatriated even if they die abroad. This allows us to have an exhaustive list including all deaths among Qatari nationals.

The Department of Psychiatry at HMC is the only provider of inpatient psychiatric care in Qatar, and it is the main provider and the only public provider of outpatient psychiatric care in the country. The number of private mental health facilities in the country is limited. Even when Qatari patients seek the services of these private facilities, they generally also have records at the Department of Psychiatry at HMC, where they can get their medication for free.

Hence, we could assume that the vast majority of people with diagnosed mental illness used the services of the Department of Psychiatry at HMC at least once.

This is, to our knowledge, the first study examining mortality in patients with mental illness in Qatar, and in the Gulf Council countries.

We aimed to confirm whether mortality is increased among patients with mental illness in Qatar, compared to those without, as this has been established in several other countries.

## Methods

We carried out a retrospective cohort study, including all Qatari nationals, deceased in 2017 and 2018, using the list of registered deaths from HMC Mortuary.

We divided the cohort into two groups: people with mental illness and those without. Subjects were included in the first group and considered to have a mental illness if they used, at least once in their lifetime, the psychiatric services at the Department of Psychiatry in HMC and received a psychiatric diagnosis and/or psychotropic medication for at least two weeks for a psychiatric indication).

For each of the groups, we collected the following variables using the electronic medical records:

- relevant sociodemographic data.
- the age at death as well as the cause of death as per the pathologist report.
- the list of psychotropic medications received over the last five years of life for at least three months.
- the last prescribed dose of antipsychotics (in chlorpromazine equivalent).
- the last height and weight (which were used to calculate the body mass index or BMI).

### Data analysis

Statistical analysis was performed using SPSS v26 (IBM Corp., Armonk, NY, USA).

For categorical variables, we determined absolute and relative frequencies. We tested continuous variables for normality using Shapiro-Wilk’s test. In case of normality, we calculated the mean and the standard deviation (SD). In case of non-normality, we calculated the median and the interquartile range (IQR).

To compare categorical variables between individuals with mental illness and those without, we used Pearson’s Chi-square and, in case of non-validity (cells with an expected count less than 5), Fischer’s exact test. We also calculated odds ratios (ORs) with their 95% Confidence intervals (95% CIs). To compare continuous variables between individuals with mental illness and those without, we used the t-test for independent samples.

To determine factors associated with age at death, we constructed a multiple linear regression model using age at death as a dependent variable, and gender, the presence of mental illness, BMI, smoking, and medical check-up during the last six months of life as independent variables.

To determine factors associated with age at death in individuals with mental illness, we constructed a multiple linear regression model using age at death as a dependent variable, with gender, smoking, use of antipsychotics, antidepressants, mood stabilizers, and benzodiazepines as independent variables.

For each of the linear regression models, the adjusted R square, the unstandardized B coefficient with its 95% CI, the partial correlation coefficient (r), as well as the p-value were calculated.

We corrected for multiple testing using Bonferroni’s method.

The defined significance level α was 0.05.

## Results

The list of registered deaths for Qatari nationals from HMC Mortuary showed 602 deaths in 2017 and 589 deaths in 2018. No medical records were found for 11 individuals (*n* = 2 for 2017 and *n* = 9 for 2018).

### Deaths in Qatari nationals in 2017 and 2018

We used published national population data to get the estimated number of Qatari individuals in 2017–2018 [17]. The mortality rate for Qatari nationals in the 2017–2018 period was calculated at 1.79 deaths per 1,000 Qataris per year. The median age at death was 65.7 years with an IQR of 31.4 years.

**Fig. 1:**
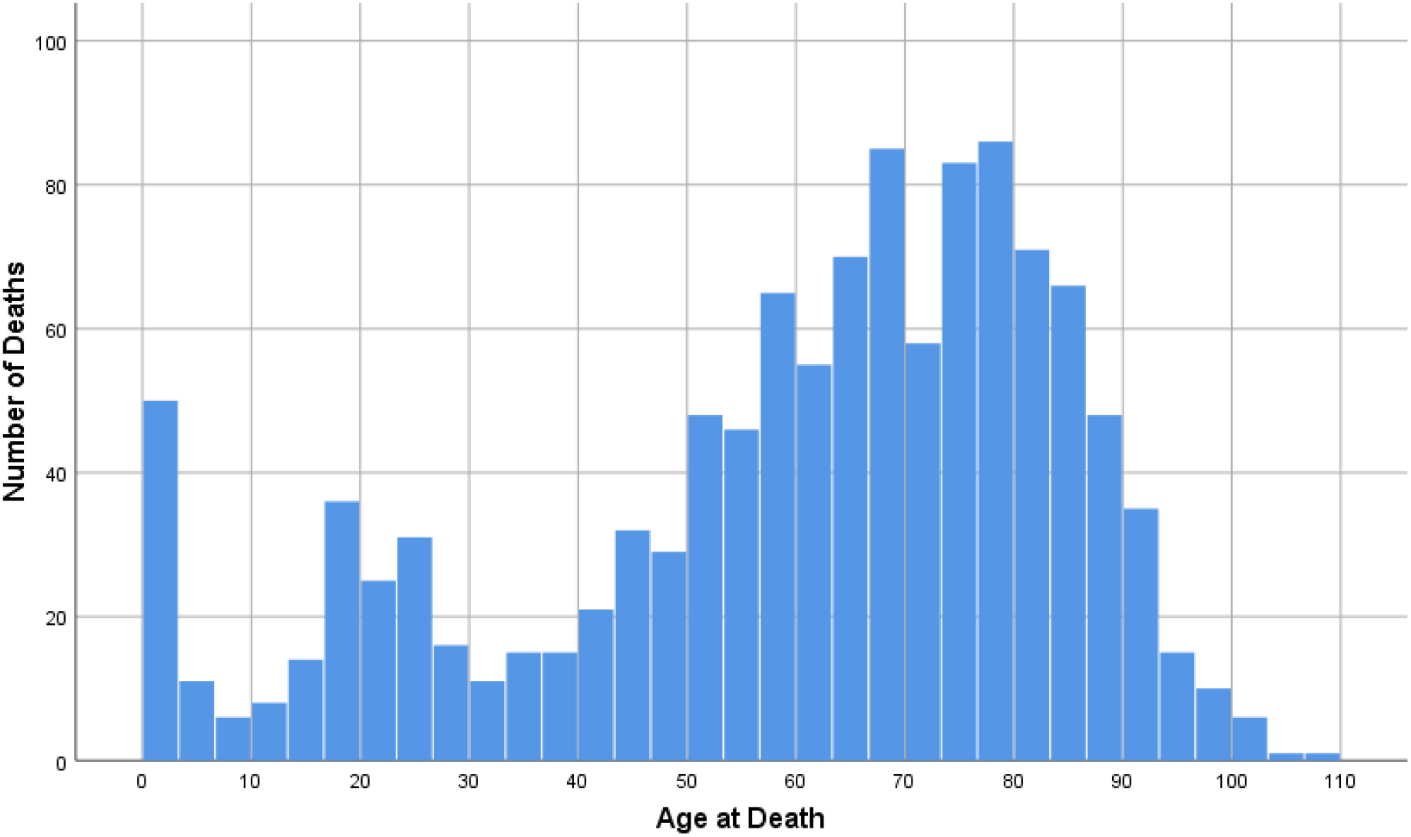
Distribution of deaths by age at death in the general Qatari population 2017–2018

Figure 1 shows the distribution of deaths by age at death.

Tables 1 and 2 show the general sociodemographic and clinical features as well as the causes of death of Qatari individuals who died in 2017 and 2018.

**Table 1:** Comparison of general sociodemographic features of Qatari individuals who died in 2017 and 2018 between those with mental illness and those without

		Without mental illness	With mental illness
Age at death (median ± IQR), in years		62.7 ± 32.9_a_	76.5 ± 22.1_b_
Gender, male, n(%)		593(63.2%)_a_	123(51.2%)_b_
Marital status, n(%)	Single	139(20.6%)_a_	28(13.5%)_b_
	Married	493(73.1%)_a_	156(75.4%)_a_
	Divorced	9(1.3%)_a_	13(6.3%)_b_
	Widow(er)	33(4.9%)_a_	10(4.8%)_a_
Smoking, lifetime, n(%)		146(26.2%)_a_	40(23.5%)_a_
Alcohol, lifetime, n(%)		28(4.4%)_a_	18(9.2%)_b_
Cannabis, lifetime, n(%)		2(0.0%)^*^	2(1.1%)_a_
Other psychoactive substances, lifetime, n(%)		2(0.3%)_a_	11(5.7%)_b_
Had a medical check-up during the last six months, n(%)		642(82.1%)_a_	210(88.6%)_b_

Comparisons are displayed using the APA style: values in the same row and subtable not sharing the same subscript are significantly different at *p* < .05

Bonferroni correction was applied to multiple comparisons

^*^This category is not used in comparisons because its column proportion is equal to zero or one

IQR: interquartile range

**Table 2:** Causes of death in Qatari nationals with and without mental illness in 2017 and 2018

Cause of death, n(%)		Without mental illness	With mental illness
Cancer		184(19.6%)_a_	34(14.2%)_a_
Cardiovascular		257(27.4%)_a_	63(26.3%)_a_
	Ischemic heart disease	102(10.9%)_a_	15(6.3%)_b_
	Stroke	30(3.2%)_a_	16(6.7%)_b_
	Other cardiovascular causes	125(13.3%)_a_	32(13.3%)_a_
Infection	Any infection	181(19.3%)_a_	77(32.1%)_b_
	Respiratory infection	69(7.3%)_a_	36(15.0%)_b_
Congenital causes		41(4.4%)_a_	2(0.8%)_b_
Chronic respiratory disease		15(1.6%)_a_	13(5.4%)_b_
Chronic Kidney Disease		18(1.9%)_a_	8(3.3%)_a_
Cirrhosis		17(1.8%)_a_	3(1.3%)_a_
Other natural causes		74(7.9%)_a_	24(10.0%)_a_
Accidental	Any accidental cause	101(10.8%)_a_	6(2.5%)_b_
	Road traffic accident	85(9.1%)_a_	3(1.3%)_b_
Suicide		2(0.2%)_a_	2(0.8%)_a_
Undetermined		49(5.2%)_a_	8(3.3%)_a_

Comparisons are displayed using the APA style: values in the same row and subtable not sharing the same subscript are significantly different at *p* < .05

Bonferroni correction was applied to multiple comparisons

^*^This category is not used in comparisons because its column proportion is equal to zero or one

### Mental illness in Qatari nationals deceased in 2017 and 2018

The prevalence of mental illness in our sample was 20.4% (*n* = 240). When selecting only subjects who died after reaching adulthood (age at death > = 18 years, *n* = 1063), the prevalence of mental illness was 22% (*n* = 234).

The most common disorders were dementia (11.5%, *n* = 135), depressive disorder (5.9%, *n* = 70), and schizophrenia (1.3%, *n* = 15) (Table 3).

**Table 3:** Most common psychiatric disorders diagnosed in Qatari subjects who died in 2017–2018

Diagnosis, n(%)	
Dementia	135(11.5)
Depressive disorder	70(5.9)
Schizophrenia	15(1.3)
Other anxiety disorders	12(1.0)
Intellectual disability	9(0.8)
Substance use disorder	8(0.7)
Bipolar disorder	6(0.5)
Adjustment disorder	3(0.3)
ADHD	3(0.3)
Personality disorder	2(0.2)
Schizoaffective disorder	2(0.2)
OCD and related disorders	1(0.1)
Schizophreniform/brief psychotic disorder	1(0.1)
Substance-induced psychotic disorder	1(0.1)

ADHD: Attention deficit hyperactivity disorder; OCD: Obsessive compulsive disorder

Antidepressants were the most common class of psychotropic drugs prescribed (46.7%, *n* = 112), followed by antipsychotics (36.7%, *n* = 88) (Table 4).

**Table 4:** Psychotropic prescriptions in Qatari subjects with mental illness who died in 2017–2018

Medication class, n(%)	
Antidepressants	112(46.7)
Mood stabilizers	12(5.0)
Antipsychotics	88(36.7)
Benzodiazepines	29(12.1)
Z hypnotics	18(7.5)
Anticholinergic drugs	15(6.3)

Compared to subjects without mental illness, subjects with mental illness had a higher age at death (median ± IQR = 76.5 ± 22.1 years vs. 62.7 ± 32.9 years; *p* < .001) and had a lower proportion of males (51.2% vs. 63.2%; *p* = .001). They were also more likely to have been single or divorced, and to have used alcohol or other psychoactive substances (Table 1).

### Causes of death in Qatari nationals with and without mental illness in 2017 and 2018 (Table 2)

Subjects with mental illness were more likely to die of an infection (OR = 1.98[1.44;2.71]), more specifically of a respiratory infection (OR = 2.23[1.45;3.42]), and of chronic respiratory disease (OR = 3.53 [1.66;7.52]) than their counterparts without mental illness. By contrast, Individuals with mental illness were less likely to die because of accidental (OR = 0.21[0.09;0.49]) or congenital causes (OR = 0.18[0.04;0.77]).

The overall proportion of deaths due to cardiovascular causes was comparable between groups (with vs. without mental illness), with ischemic heart disease being a less common cause of death (OR = 0.55[0.31;0.96]) and stroke being a more frequent cause of death in subjects with mental illness (OR = 2.16[1.16;4.04]).

No differences were found between Qataris with and those without mental illness in the proportion of deaths by cancer or suicide.

Factors associated with age at death in Qatari nationals (whole cohort, with and without mental illness) deceased in 2017 and 2018 (Table 5).

**Table 5:** Factors associated with age at death in Qatari nationals deceased in 2017 and 2018

				95%Confidence Interval for B		Partial Correlation Coefficient
Variable		B	p	Lower Bound	Upper Bound	
	Mental illness	6.691	0.000	3.464	9.918	0.161
	Gender	0.191	0.906	-2.969	3.351	0.005
	Smoking	-4.327	0.016	-7.846	− 0.809	− 0.097
	BMI	− 0.163	0.055	− 0.330	0.003	− 0.077
	Medical check-up during the last six months of life	13.362	0.000	8.510	18.215	0.212

In multiple regression analysis, we found that higher age at death was associated with the presence of mental illness (B = 6.691[3.464,9.918], *r* = .161, *p* = .000), with the absence of smoking B=-4.327[-7.846,-0.809], *r*=-.097, *p* = .016), and with the presence of medical check-up during the last six months of life (B = 13.362[8.510,18.215], *r* = .212, *p* = .000). Gender and BMI were not associated with age at death.

### Factors associated with age at death in Qatari nationals with mental illness deceased in 2017 and 2018 (Tables 6 and Fig. 2)

**Table 6:** Factors associated with age at death in Qatari nationals with mental illness deceased in 2017 and 2018

				95% Confidence Interval for B		Partial Correlation Coefficient
Variable		B	p	Lower Bound	Upper Bound	
	Gender	-2.383	0.484	-9.094	4.328	− 0.055
	Smoking	-7.881	0.058	-16.020	0.258	− 0.148
	Antipsychotic use	-3.848	0.239	-10.274	2.579	− 0.092
	Antidepressant use	4.179	0.181	-1.968	10.325	0.105
	Mood stabilizer use	− 0.791	0.908	-14.231	12.648	− 0.009
	Benzodiazepine use	1.389	0.767	-7.846	10.624	0.023

**Fig. 2:**
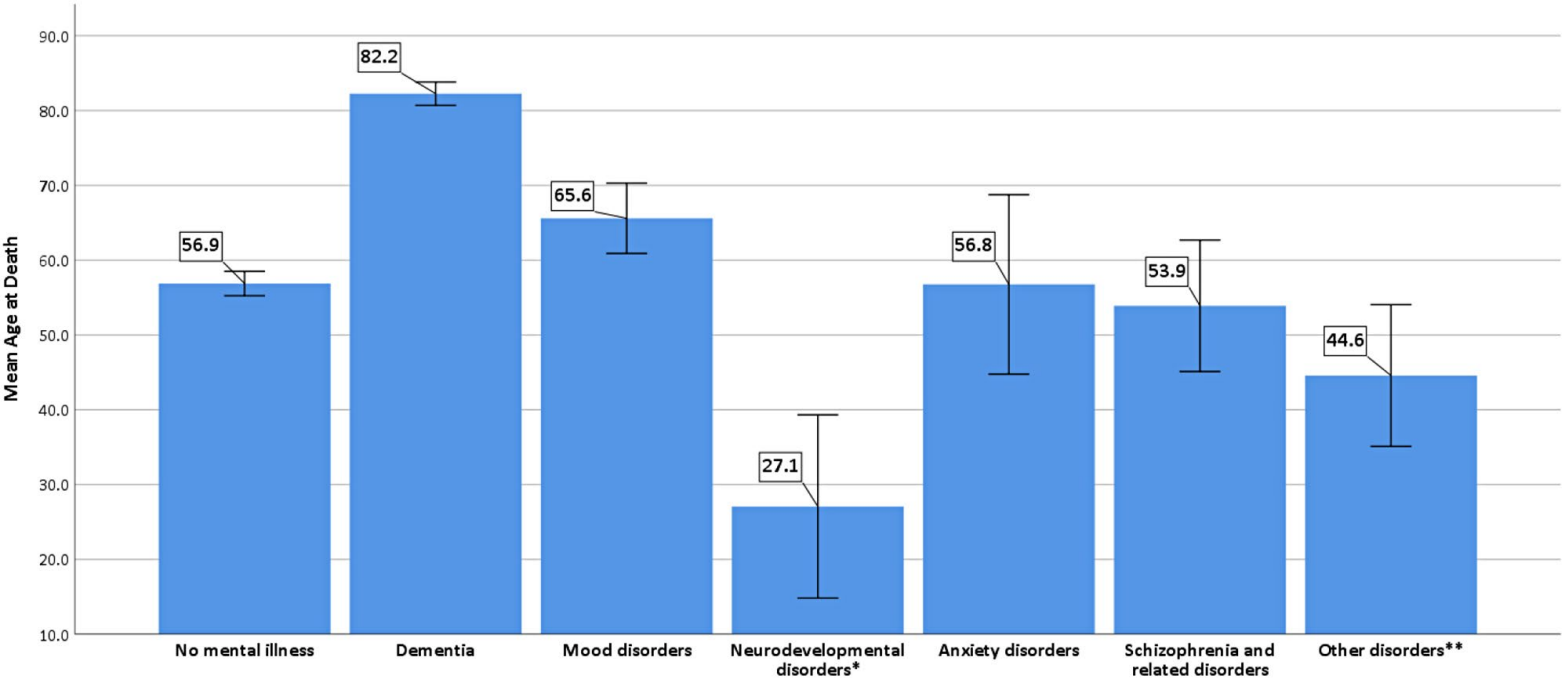
Mean age at death for the different categories of psychiatric disorders compared to those without mental illness. * Includes Attention deficit hyperactivity disorder and Intellectual disability. ** Includes personality, substance-related, and stress-related disorders

In multiple regression analysis, we did not find gender, smoking, or use of any class of psychotropics to be associated with age at death in individuals with mental illness (Table 6).

In subjects on antipsychotic medication (*n* = 92), higher prescribed chlorpromazine-equivalent doses were significantly associated with lower age at death (B=-0.036[-0.059,-0.013], *r*=-.387, *p* = .002) after controlling for gender and smoking. This association remains significant even after controlling for other major cardiovascular risk factors (diabetes, hypertension, and dyslipidemia).

Compared to individuals without mental illness (mean age at death = 56.9 years [55.2;55.2]), age at death was higher in patients with dementia (82.2 years [80.7;80.7], adjusted *p* < .001) and lower in subjects with intellectual disability and/or ADHD 27.1[14.8;14.8], adjusted *p* = .003). Age at death for mood disorders, schizophrenia and related disorders, anxiety disorders and other psychiatric disorders was not significantly different from that of subjects without mental illness (Fig. 2).

## Discussion

In this retrospective cohort study, we found that subjects with mental illness surprisingly had a higher mean age at death on average than those without mental illness. This difference persisted even after we controlled for covariates (gender, smoking, BMI, medical check-up during the last six months of life as a proxy for regular medical screenings/follow-ups).

### Prevalence of mental illness in Qatari individuals deceased in 2017 and 2018

We found that 22% of Qatari people who passed away at or after the age of 18 had a diagnosed mental illness. A previous population-based epidemiological study found that the most common mental disorders in the Qatari adult population (18–65 years) were generalized anxiety disorder (20.4%), and major depression (19.1%) [18]. The prevalence of mental illness in our study might thus be slightly underestimated. One possibility is that our definition of mental illness (going through HMC records) might have made us miss a number of psychiatric diagnoses which were exclusively treated in the private sector or abroad without being mentioned on the participants’ medical records. However, it is also likely that the difference was due to an underdiagnosis of psychiatric disorders mainly because of social stigma among other factors [19]. In addition to social stigma, religious stigma and fear of legal consequences affect the reporting of substance use. In a published epidemiological survey from Qatar, all participants denied use of substances and ever having suicidal ideations. This probable underdiagnosis mainly affected “milder” psychiatric disorders like anxiety disorders (for which we found a prevalence of 1% compared to 20.4% for generalized anxiety disorder alone reported in a previous epidemiological study in Qatar) [18].

### Mortality in Qatari subjects with mental illness

Our finding that subjects with mental illness had a higher mean age at death on average than subjects without mental illness is totally unexpected. This difference does not seem to be explained by any disparities in gender, smoking, BMI, or regular medical screenings/follow-ups. This result is in stark contrast with the overwhelming evidence that has associated mental illness with higher mortality. Walker et al.’s systematic review and meta-analysis reported that out of the included 148 studies, the vast majority (91.2%, *n* = 135) found that people with mental disorders had a significantly higher mortality than controls. In the same study, the remaining 9.4% (*n* = 14) had negative findings, and no studies reported a lower mortality in individuals with mental illness. Overall, the pooled relative risk of all-cause mortality was 2.22 [3]. A more recent meta-analysis that focused on people with severe mental illness also reported pooled relative risks for all-cause mortality of 2.89 in schizophrenia and 2.51 in bipolar disorder [20]. There has been no evidence that this gap is narrowing over the years [20, 21], and the gap might even be widening [3].

One of the possible explanations for our result is that the incidence of dementia clearly increases with age, and individuals who died older are more likely to have been diagnosed with dementia than individuals who died younger [22]. However, even individuals with psychiatric disorders other than dementia did not seem to exhibit higher mortality.

Previous studies found increased mortality in people with schizophrenia [4, 7, 20], schizoaffective disorder [7], bipolar disorder [7, 8, 20], unipolar depression [7, 10], acute psychotic disorder [6], as well as anxiety disorders [11]. In the meta-analysis by Walker et al., mortality was significantly higher for psychotic disorders than for depression, bipolar disorder, or anxiety [3].

Several factors were traditionally reported to explain the higher overall mortality in subjects with mental illness. These include deaths from “unnatural causes”, poorer lifestyle, a higher prevalence of metabolic syndrome, access to care difficulties, poorer quality of care received for any medical comorbidities, as well as potential iatrogenic complications [3, 20, 21, 23].

In the Qatari population, as our findings suggest, deaths from suicide seem to be extremely rare both in people with and in people without mental illness. This is consistent with a previous study that examined the 20-year trend of age‑standardized suicide rates in 46 Muslim-majority countries using the WHO Global Health Estimates. The study revealed that suicide rates in the studied countries, including Qatar, were low compared to the global average rates [24].

In contrast with other studies from Western countries, people without mental illness seem to be at a higher risk of accidental death than those with mental illness. These deaths are mostly due to road traffic accidents and occur at a young age. In particular, young male drivers (often defined as below 25 years of age) in Qatar had a relative risk for road mortality 10 times higher than the general population [25]. Several factors contributing to this extremely high mortality were highlighted: use of mobile phones while driving, risky driving habits and traffic law violations [25, 26]. These young drivers generally have no underlying psychiatric disorder and die young thus decreasing their lifetime prevalence of mental illness.

While smoking is often reported to be more common in people with mental illness in Western countries than in people without, with this gap widening over time [27], this does not seem to be necessarily the case in the Qatari population. Indeed, in the present study, the prevalence of lifetime smoking was comparable in both groups (around one in four). These figures are not very different from those reported by an epidemiological study of tobacco use in Qatar (current use in 20.6% of Qataris) [28].

Moreover, even though we found that subjects with mental illness were more likely to have used alcohol or other psychoactive substances as previously reported in other parts of the world [21], the prevalence of use of these substances remained relatively low in our study. This is probably due to sociocultural and religious factors, and may contribute to explaining why Qatari subjects with mental illness did not seem to exhibit higher mortality.

A high prevalence of obesity and metabolic syndrome are also factors contributing to the excessive mortality in individuals with mental illness, mainly through an increase in mortality due to cardiovascular disease [3, 20, 21]. However, we found that the proportion of deaths due to cardiovascular causes was not significantly different between groups. This might seem unexpected given that previous studies worldwide [29–31] and in Qatar [32, 33] clearly showed a high prevalence of metabolic syndrome and a high cardiovascular risk in patients with mental illness.

One possible explanation is that sedentary lifestyle, obesity and metabolic syndrome are very common in Qatar in the general population [15]. Indeed, a previous study found that the prevalence of metabolic syndrome was similarly high in patients on antipsychotics and in healthy controls in Qatar [16]. Another study showed that drug-free patients with bipolar disorder or schizophrenia did not differ much from healthy controls in terms of metabolic syndrome components [34].

Poor access to healthcare mainly driven by poverty, stigma, as well as certain disabling symptoms of mental illness (cognitive, negative, or depressive symptoms) is often cited as one of the factors explaining premature death in psychiatric patients [21]. However, this is less likely to apply to Qatar, where healthcare is available for free to all citizens, and where community healthcare services are increasingly provided throughout the country. High standards of living and strong family support among Qataris can also improve access to healthcare. In the present study, we found that in fact individuals with mental illness were more likely to have had a medical check-up over the last six months of their lives. This highlights the important role psychiatrists can play in screening for, initiating and coordinating the management of physical health conditions in their patients [35].

Having access to healthcare is one thing and receiving the same standard of care is another. An extensive body of research has highlighted the disparities in terms of quality of physical healthcare between patients with mental illness and those without [36–40]. There is evidence that such a disparity also exists in Qatar [39] as well as in other Gulf Council countries [41]. Our findings, however, do not seem to corroborate these previous results, in the absence of excessive mortality and the seemingly more frequent medical check-ups in individuals with mental illness. One possible explanation is that the aforementioned studies compared groups diagnosed with a physical condition, with one having a comorbid mental illness and the other one without; whereas in the present study, the control group included all individuals who passed away in 2017 and 2018, even those without an established diagnosis of a physical condition. It seems plausible that psychiatric patients might benefit from more frequent screening of the most common medical comorbidities thanks to their mental health check-ups. These conditions might, hence, be recognized earlier than in the general population where they can remain asymptomatic and unrecognized for years [42]. However, once both groups are diagnosed with the same physical condition, individuals with mental illness might be at a disadvantage.

Iatrogenic factors have also been incriminated in the excessive mortality of patients with mental illness worldwide, most notably due to metabolic and cardiovascular side effects [29]. However, these complications have mainly been attributed to antipsychotics and only one third of the subjects with mental illness in this study received antipsychotic medication.

### Unnatural causes of death in Qatari nationals with and without mental illness in 2017 and 2018

Unlike previous findings, mostly from Western countries, where unnatural deaths accounted for roughly one third of the causes of death in patients with mental illness [3], we found that unnatural death represented less than 5% of the causes of death in the present study.

This is probably largely due to very low numbers of suicide: as low as two cases in each group (with and without mental illness). These low rates of suicide in Muslim-majority and in Arab countries in general, and in Qatar in particular, can be partly explained by the role of religious beliefs in decreasing suicidal behavior, not only because Islam strongly condemns suicide, but also because it can confer a particular meaning of life to its adherents, and provide them with a certain perspective when going through difficult periods of their lives. In addition, similarly to other Muslim and Arab societies, the Qatari society has mostly collectivist norms, and is characterized by strong family and community support, which can be protective factors against suicide [24, 43]. The literature about suicide in Muslim-majority countries is scarce, and many previous studies suspected that suicide is likely underreported in these countries due to social stigma [24, 43, 44]. Nonetheless, in the present study, a forensic report was available for each case and the proportion of “undetermined causes of death” was rather low. The latter proportion was also comparable between subjects with and those without mental illness, thus leading us to believe that the suicide rates were actually very low in both groups.

Accidental deaths were also very rare (2.5%) in individuals with mental illness in the present study, even lower than in controls. This is also in stark contrast with a meta-analysis showing that patients with schizophrenia and those with bipolar disorder had a pooled relative risk for accidental death of about 6 and 3.5 respectively [20]. A Swedish national cohort study showed that all mental disorders were strong risk factors for accidental death [45]. It is plausible that the low proportions of substance use partly explains the low risk of accidental death in Qatari patients with mental illness. Nonetheless, substance use is not the only factor explaining excessive accidental mortality in individuals with mental illness. Other factors are related to the condition itself (reckless behavior, fatigue, sleep disturbance, and cognitive dysfunction causing increased reaction times) and to the psychotropic medication (sedation, cognitive side effects) [45].

### Natural causes of death in Qatari nationals with and without mental illness in 2017 and 2018

Our findings suggest that the most common causes of death in Qatari subjects with mental illness are infections followed by cardiovascular disease then cancer. Qatari Individuals with mental illness were more likely to die of an infection or of a chronic respiratory condition, but as likely to die from cancer or cardiovascular disease as controls.

In most previous research, mental illness was associated with a higher risk of death from cardiovascular disease, cancer, infections, chronic respiratory conditions and most other natural causes of death [3, 20, 46–49].

Factors explaining the typically excessive cardiovascular mortality in people with mental illness include smoking, poor lifestyle, and poorer quality of care, antipsychotic medication, as well as possible genetic links between certain psychiatric disorders and the metabolic syndrome [1, 3, 50, 51].

In the present study, cardiovascular mortality was not found to be increased in people with mental illness. This can be explained by a relatively low proportion of smokers, and by a likely narrower treatment gap with individuals without mental illness (given that all Qataris enjoy free and easy-to-access healthcare services across the country).

It is not very clear why Qatari individuals with mental illness do not seem be more likely to smoke than those without. It is possible that sociocultural factors play a role. Moreover, smoking in subjects with mental illness has been shown to be especially high in people with severe mental illness, which only represent a minor fraction of psychiatric disorders in our study. The link between smoking and “less severe” psychiatric disorders including depressive and anxiety disorders, although likely present, seems to be less strong [52].

Our findings may suggest that when patients with mental illness have easy access to mental health care, their regular check-ups with their psychiatrists can be good opportunities to screen for and initiate the management for any comorbid medical conditions like diabetes, dyslipidemia, or hypertension. This role, if properly carried out by mental health professionals, may help decrease not only cardiovascular mortality [35], but also mortality from other medical conditions that can benefit from regular screening, like cancer [53]. This is also possibly on of the reasons why the present study did not show that patients with mental illness had excessive mortality due to cancer.

In the present study, the first cause of death in people with mental illness was infection. Many psychiatric disorders, including depression [54], stress-related disorders [55] and schizophrenia [49], have been associated with increased incidence of various infections, especially severe ones. Apart from individuals with mental illness being more likely to smoke and to have a low socioeconomic status, immune dysregulation processes [56], epigenetic mechanisms [55], and iatrogenic factors [57] also play a role in this association.

In Qatar, since smoking and socioeconomic differences cannot probably explain the difference between people with mental illness and people without (as detailed above) in terms of mortality due to infections, it is likely that the epigenetic, immune, and iatrogenic factors play a more important role. Disparities in the quality of care cannot be excluded, but need further studies to be confirmed [21].

In the present study, respiratory infections accounted for almost one half of the infections reported as a cause of death in patients with mental illness. This is also in line with deaths from chronic respiratory conditions being higher in Qatari subjects with mental illness than in those without. Previous studies have linked different psychiatric disorders (including mood and anxiety disorders as well as schizophrenia) to chronic obstructive pulmonary disease (COPD) and asthma [49, 58–60]. Depression and anxiety can increase the number of COPD exacerbations, and the length of hospital stay [60]. More importantly, depression and anxiety were shown to increase the risk of COPD mortality by 1.83 and 1.27 respectively [59]. In addition, antipsychotics have been linked to a dose-dependent increased risk of acute respiratory failure in COPD patients [61].

### Factors associated with age at death in Qatari nationals with mental illness deceased in 2017 and 2018

In patients on antipsychotics, higher antipsychotic doses were significantly associated with lower age at death. The role of antipsychotics in the premature death observed in patients on antipsychotic medication has been debated with different studies yielding conflicting findings [62–64].

It is possible that antipsychotics have some protective and other detrimental effects on mortality explaining the U-shaped curve that the cumulative dose of antipsychotics exhibits for all-cause mortality [65]. Untreated patients might be at a higher risk of mortality due to the symptoms and complications of their underlying mental illness. At the same time, high doses of antipsychotic medication may increase the propensity for certain side effects that are likely dose-dependent [63, 65, 66]. In particular, cardiovascular effects including QTc prolongation and autonomic side effects may increase with the dose prescribed, and contribute to excessive cardiovascular mortality [62, 63].

The direction of the link between the antipsychotic dose and mortality can be seen from a different angle. It is possible that individuals with mental illness at a higher risk of mortality tend to have a severe, difficult-to-treat illness that may require higher doses of antipsychotics [63, 65, 66].

## Strengths and limitations

To the best of our knowledge, this study is the first to examine mortality in patients with mental illness in Qatar, and in the Gulf Council countries, and is one of very few studies from the Arab World and the Middle East. We were able to exhaustively include all Qataris who passed away in 2017 and 2018. In addition, thanks to the fact that HMC is by far the largest provider of mental health care in the country, we believe we were probably able to capture most diagnoses of mental illness.

Nonetheless, a few limitations are to be noted. It is still possible that due to underdiagnosis and/or treatment abroad, certain psychiatric diagnoses were not adequately captured in the study. Moreover, given the retrospective nature of the study, the quality of our data heavily depended on the quality of the medical records.

## Conclusions

In contrast with most previously published studies (mainly from Western countries), we did not find that mortality was higher in Qatari individuals with mental illness in comparison to those without. Sociocultural factors might have played a significant role in decreasing the use of tobacco, alcohol, and other psychoactive substances. Religious and social factors also probably explain a low incidence of suicide. Free and easy-to-access healthcare for all citizens might have improved the “classical” quality of care disparities between those with mental illness and those without. Finally, the role of mental health services in screening for and initiating the management of physical conditions may even reverse the “usual trend” of increased mortality in patients with psychiatric disorders.

## Data Availability

The datasets used and/or analyzed during the current study are available from the corresponding author on reasonable request, pending approvals from Hamad Medical Corporation.
